# Supplementary material for: Predation and fragmentation portrayed in the statistical structure of prey time series
Source: BMC Ecol. 2009 May 6;9:10. doi: 10.1186/1472-6785-9-10 (PMC2689204; doi:10.1186/1472-6785-9-10)
Supplement: Additional file 2 — Voles and related classes ODDox Documentation. ODDox documentation of the agent-based model (ALMaSS) applied by Hendrichsen et al. The documentation is started by activating main.html. [file 1472-6785-9-10-S2.zip › Vole_ODDox/_boost_random_generators_8h.html]

ALMaSS ODDox: BoostRandomGenerators.h File Reference

- Main Page
- Related Pages
- Classes
- Files

# BoostRandomGenerators.h File Reference

---

## Detailed Description

**BoostRandomGenerators.h Boost headers for using the boost random number generation utilities**

`#include <ctime>`  
`#include <boost/random/linear_congruential.hpp>`  
`#include <boost/random/uniform_int.hpp>`  
`#include <boost/random/uniform_real.hpp>`  
`#include <boost/random/variate_generator.hpp>`  
`#include <boost/random/lagged_fibonacci.hpp>`  
`#include <boost/generator_iterator.hpp>`  

|  |  |
| --- | --- |
|  |  |
| Typedefs | |
| typedef   boost::lagged\_fibonacci19937 | base\_generator\_type |
| typedef boost::uniform\_int | distribution\_type |
| typedef   boost::variate\_generator  < base\_generator\_type   &, distribution\_type > | gen\_type\_int |

---

## Typedef Documentation

typedef boost::lagged\_fibonacci19937 base\_generator\_type

typedef boost::uniform\_int distribution\_type

typedef boost::variate\_generator<base\_generator\_type&, distribution\_type> gen\_type\_int

---

Generated on Thu Jan 22 14:13:45 2009 for ALMaSS ODDox by 
 1.5.6
